# A sex allocation cost to polyandry in a parasitoid wasp

**DOI:** 10.1098/rsbl.2015.0205

**Published:** 2015-06

**Authors:** Rebecca A. Boulton, David M. Shuker

**Affiliations:** School of Biology, University of St Andrews, St Andrews, UK

**Keywords:** polyandry, sex allocation, sexual conflict

## Abstract

The costs and benefits of polyandry are central to understanding the near-ubiquity of female multiple mating. Here, we present evidence of a novel cost of polyandry: disrupted sex allocation. In *Nasonia vitripennis*, a species that is monandrous in the wild but engages in polyandry under laboratory culture conditions, sexual harassment during oviposition results in increased production of sons under conditions that favour female-biased sex ratios. In addition, females more likely to re-mate under harassment produce the least female-biased sex ratios, and these females are unable to mitigate this cost by increasing offspring production. Our results therefore argue that polyandry does not serve to mitigate the costs of harassment (convenience polyandry) in *Nasonia*. Furthermore, because males benefit from female-biased offspring sex ratios, harassment of ovipositing females also creates a novel cost of that harassment for males.

## 1. Introduction

Explaining the origin and maintenance of polyandry is key to understanding the evolution of mating systems, patterns of sexual selection and the role of sexual conflict [1]. In recent years, it has become clear that females of many species gain direct benefits from low to intermediate levels of polyandry [2], benefits that can offset the costs of mating. Alternatively, females may become polyandrous in order to limit male harassment, whereby re-mating is less costly than trying to reject and avoid persistent males, an outcome termed ‘convenience polyandry’ [3]. However, much of this work has focused on species that already are polyandrous (often for sensible logistical reasons). We have recently begun to explore the costs and benefits of polyandry in the parasitoid wasp *Nasonia vitripennis*, a species that is ‘mostly monandrous' in the wild [4], albeit with segregating variation in re-mating rate and which evolves greater polyandry under laboratory culture [5].

Here, we consider a novel cost of polyandry that emerges from sex allocation in *N. vitripennis. Nasonia vitripennis* is haplodiploid, and so inseminated females can produce both haploid sons (from unfertilized eggs) and diploid daughters (from fertilized eggs). Females allocate sex in line with the predictions of local mate competition (LMC) theory, producing a very female-biased sex ratio when ovipositing alone [6,7]. Although our recent work suggests that mating with virgin males provides females with a direct fecundity benefit, there is also evidence to suggest that mating and/or harassment during oviposition disrupts facultative sex allocation, resulting in a less female-biased sex ratio [8]. There are at least two possible non-adaptive explanations for this phenomenon: first, if exposure to males increases female mating rate (i.e. polyandry), then repeated inseminations may limit sperm use for sex allocation (females are less able to mobilize sperm for up to 24 h when multiple males inseminate in close succession: [8]). Second, male interference by itself during oviposition may disrupt female ability to fertilize eggs [9]. Understanding whether or not increased mating and/or harassment imposes a sex allocation cost is important for examining whether convenience polyandry might be a beneficial strategy for female *N. vitripennis* [4]. If repeated matings replace one cost (of harassment) with another (reduced ability to use sperm), then polyandry is unlikely to be convenient enough for female *Nasonia*.

Here, we extend our previous study [4], experimentally manipulating female receptivity to further mating by varying female exposure to male post-copulatory courtship following an initial mating. Females are more likely to accept further mating attempts if they have not experienced post-copulatory courtship ([8]; see below), and so this will increase the level of polyandry. We then explored the costs of mating and harassment in terms of facultative sex allocation under LMC. If exposure to males during oviposition results in a sex allocation cost, females that are rendered more likely to re-mate by preventing post-copulatory courtship should then suffer the most under harassment, producing a less female-biased sex ratio than resistant females. If, however, polyandry is convenient, we predict that the sex allocation cost of harassment will be mitigated by increased oviposition time gained by reducing male harassment. As such, females that re-mate under harassment should lay more eggs in total, even if a smaller proportion are daughters.

## 2. Material and methods

The strain of *N. vitripennis* used was HVRx, maintained as a large outbred population [10]. Males perform a stereotyped pre- and post-copulatory courtship display, the former of which serves to initiate receptivity and the latter to reduce female receptivity to future mating attempts [11]. In order to investigate whether females that re-mate under harassment gain a reproductive advantage, we allowed females to oviposit on six *Calliphora vicina* blowfly pupae (‘hosts') for 24 h either: (i) in the presence of five males (harassment: H) or (ii) alone (control: C). All of these females had mated once 24 h prior to the addition of hosts. After this initial mating, half of the females were allowed to experience post-copulatory courtship (P) and half were not (NP). In the latter case, we prevented post-copulatory courtship by moving the male away using a paintbrush after copulation. The factorial design comprised four treatments in total: (i) no post-copulatory courtship, no harassment (NPC); (ii) no post-copulatory courtship, harassment (NPH); (iii) post-copulatory courtship, no harassment (PC); and (iv) post-copulatory courtship, harassment (PH). In the two H treatments, after males and hosts were provided, the females were observed until a male attempted courtship. We then scored whether the female refused or accepted the copulation. For data analysis, we used a general and generalized linear modelling (GLM) approach implemented in R.

## 3. Results

**Figure 1.**
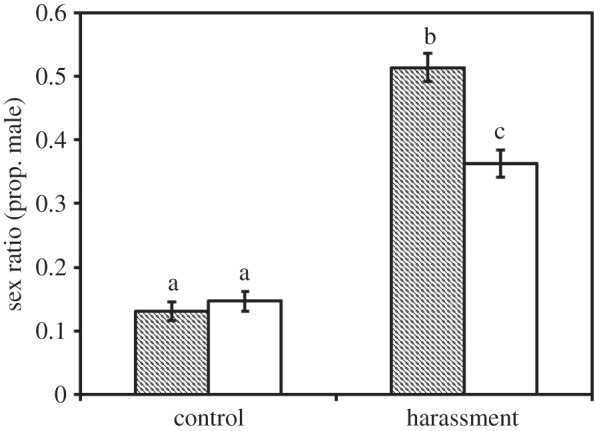
Offspring sex ratios (proportion of offspring that were male) produced by females that experienced harassment during oviposition versus control females that did not. Females also either did (open bars) or did not (filled bars) experience post-copulatory courtship after their initial mating. Lowercase letters indicate significant differences between treatments. Error bars are binomial CIs.

Post-copulatory courtship after a female's initial mating reduced the likelihood of mating with the first male that courted 24 h later when that female was exposed to both males and hosts (42% versus 81%; quasi-binomial GLM: *F*_1,87_ = 14.94, *p* < 0.001). The presence of males during oviposition significantly influenced sex allocation: females that experienced harassment produced a significantly less female-biased sex ratio than control females (*F*_1,194_ = 78.62, *p* < 0.0001; figure 1). Importantly, post-copulatory courtship itself also influenced sex allocation, with NP females producing a less female-biased sex ratio than P females (*F*_1,194_ = 4.27, *p* = 0.04). The interaction between harassment and post-copulatory courtship was marginally non-significant (*F*_1,192_ = 3.86, *p* = 0.051), and there was no effect of post-copulatory courtship on sex ratio in the control conditions (NPC versus PC: pairwise *F*_1,90_ = 0.17, *p* = 0.68).

**Figure 2.**
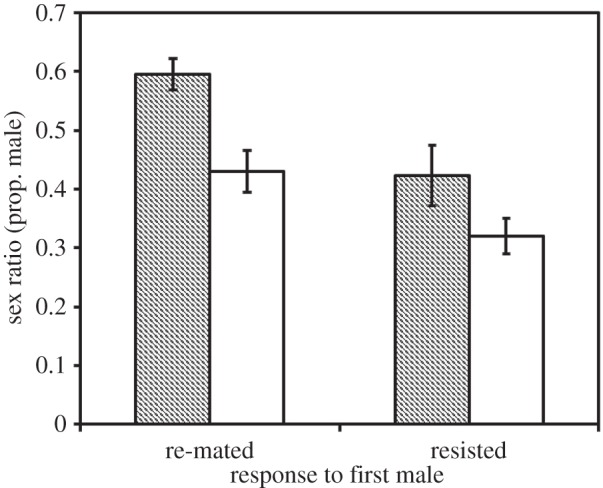
Offspring sex ratio (proportion of offspring that were male) produced by females that either re-mated or resisted the first male that courted them when exposed to males in the harassment treatments. Females either did (open bars) or did not (filled bars) experience post-copulatory courtship after their initial mating. Error bars are binomial CIs.

Females observed to re-mate with the first male that courted them (under harassment) laid a less female-biased sex ratio than females that resisted (although this was only marginally significant: *F*_1,84_ = 4.02, *p* = 0.048; figure 2). In addition, among females which had the opportunity to re-mate, those that did not receive post-copulatory courtship produced less female-biased sex ratios as before (*F*_1,87_ = 5.06, *p* = 0.03), and the effects of observed re-mating and post-copulatory courtship were again independent of each other (interaction: *F*_1,87_ = 0.11, *p* = 0.74; figure 2).

While there was a reduction in daughter production in the presence of males during egg laying (*F*_1,194_ = 46.86, *p* < 0.0001), the presence of males did not influence total offspring production (*F*_1,194_ = 0.09, *p* = 0.76). This was the case whether females experienced post-copulatory courtship or not (*F*_1,194_ = 0.63, *p* = 0.43). Moreover, polyandry did not appear to allow more time to oviposit, as females observed to re-mate did not lay more eggs (*F*_1,83_ = 0.02, *p* = 0.90) regardless of post-copulatory courtship (interaction: *F*_1,83_ = 1.92, *p* = 0.17). Furthermore, there was no correlation between sex ratio and offspring production for females observed to re-mate (*r*_50_ = −0.001, *p* = 0.99).

## 4. Discussion

The presence of males during oviposition leads to disrupted sex allocation for female *N. vitripennis*. Females lay more sons in the presence of males and fail to produce the extremely female-biased sex ratios characteristic of female *Nasonia* under conditions of high LMC [6,7,12]. Whether or not females experienced post-copulatory courtship after their first mating was also associated with this effect, suggesting that male courtship and mating may influence a female's capacity to fertilize her eggs. Females that did not receive post-copulatory courtship were twice as likely to accept a second mating at the first opportunity, and they also produced less female-biased sex ratios than females which experienced post-copulatory courtship. This implies that disrupted sex allocation is associated with polyandry in *N. vitripennis*. Our results also strongly suggest that extra inseminations influence female ability to use sperm for daughter production in *N. vitripennis* [4].

Females which experienced harassment produced less female-biased sex ratios (around 0.3) than the control females (around 0.1) which had no opportunity to re-mate and were not disturbed during egg laying. This occurred regardless of whether females under harassment re-mated or experienced post-copulatory courtship. Previous work suggests that physical disturbance during (but not before) oviposition can disrupt sex allocation [4,9], although there is no evidence that the presence of males is used by females as an LMC cue. Our findings instead suggest that harassment may carry its own sex allocation cost independently of re-mating, but we cannot rule out that females which initially resisted did later re-mate (potentially multiple times).

These findings may represent something of a paradox, however, for males. Due to haplodiploidy, males only achieve fitness through daughters [13]. This means that by overcoming female resistance and ‘winning’ the pre-copulatory sexual conflict over mating, males then lose out in post-copulatory sexual conflict over sex allocation, as daughter production (fertilization) is lowered. In the current experiment, only one non-virgin female was available, and having some paternity success is obviously better than having none at all. However, by inhibiting female sperm use in general, second males are reducing the number of eggs available to fertilize. It may be that sperm blocking increases the relative paternity of second males. Sperm-depleted males of the parasitoid *Trichogramma euproctidis* appear to increase their relative paternity by continuing to mate, as this reduces females' ability to store sperm from subsequent matings [14]. Any such effect in sperm-competent *Nasonia* would have to overcome the direct cost of reduced sperm use.

Further study into the mechanistic basis of this novel sex-allocation cost of multiple mating may therefore help to shed light on any adaptive value of males re-mating with recently mated females, which appear to reduce their mates' daughter production as well as that of their rivals. In other insect species, interaction between ejaculate components can result in incapacitation or displacement of rival male sperm [15]. We currently know very little about ejaculate composition in *N. vitripennis* and there is no genomic evidence for the presence of accessory gland proteins that are found in other insects [16]. It therefore remains to be seen what causes female sperm use to be delayed following multiple mating, and whether it is adaptive for males and/or for females.

If female *Nasonia* do allow re-mating in the face of male harassment, this does not lead to females producing more offspring. Females are therefore not trading-off higher offspring production with lower daughter production (i.e. producing more offspring, but a higher proportion of sons). Instead, females are only paying a sex allocation cost. As such, polyandry does not appear to be convenient for female *N. vitripennis* when LMC is intense. Perhaps, the most salient point that can be made about this sex allocation cost of polyandry and harassment is that it is context dependent. Under conditions of reduced LMC, where multiple females parasitize the same host(s) (superparasitism), females should produce sex ratios approaching equality to maximize their fitness. Under low LMC, the sex allocation ‘cost’ of polyandry and harassment will thus be reduced. Superparasitism is typical in standard laboratory culture conditions and may occur naturally when host patches are large enough to allow exploitation by multiple females. We may thus expect that mating patterns, and consequently sexual selection, will be closely related to habitat structure in *N. vitripennis*.

## Data accessibility

All data are available through the Dryad data repository: http://dx.doi.org/10.5061/dryad.qb8vf.
